# Commentary: Methamphetamine abuse impairs motor cortical plasticity and function

**DOI:** 10.3389/fnhum.2017.00562

**Published:** 2017-11-24

**Authors:** Xiangju Du, Chang Yu, Zhen-Yu Hu, Dong-Sheng Zhou

**Affiliations:** Ningbo Kangning Hospital, Ningbo, China

**Keywords:** addiction, schizophrenia, depression, TMS, NIBS

Psychiatric diseases demonstrate plasticity deficits in the brain. Animal studies have investigated the topic extensively. For instance, brain slice experiments with hippocampus/cortex preparations revealed plasticity changes in synaptic transmission of certain pathways, in a line with the learning and memory impairments in certain psychiatric diseases (Duman et al., 2016). Addiction is associated with synaptic transmission changes in mesolimbic and mesocortical pathways, with alterations of synaptic plasticity reported (Lüscher and Malenka, 2011). With an arsenal of animal reports on addiction evoked brain plasticity, surprisingly there were few studies translating such findings onto human subjects (Etkin, 2016). In a recent study published on the journal of *Molecular Psychiatry*, Huang et al. heroically investigated the cortical functional changes following methamphetamine abuse both in animal model and human addicts (Huang et al., 2017).

The authors firstly set up the animal model of methamphetamine self-administration and examined the synaptic plasticity on brain slices. The results showed that motor cortical, and dorsal-lateral rather than dorsal-medial striatal pathways exhibited impaired plasticity induction. Interestingly, molecular expression of GluN3A-containing NMDA receptors seems to be attributed for the altered plasticity. This is in a line with the previous finding that insertion of GluN3A-containing NMDA receptors at midbrain dopamine neurons resulted in anti-hebbian like plasticity (Mameli et al., 2011), given the fact that these NMDA receptors are less calcium permeable than canonical NMDA receptors.

To correlate the animal findings with human cortical plasticity, the authors employed a surrogate of synaptic plasticity in human—the plasticity of transcranial magnetic stimulation (TMS)-induced motor evoked potential (MEPs) (Huang et al., 2005), to dissect the potential impacts of methamphetamine on motor cortex. Notably, the Long-term potentiation (LTP) or Long-Term depression (LTD)-like changes of MEPs were both impaired in methamphetamine abusers, indicating that the cortical plasticity is impaired in human addicts. Interestingly, the plasticity deficits were in parallel with motor learning impairments, both in animal and human subjects (Figure 1).

**Figure 1:**
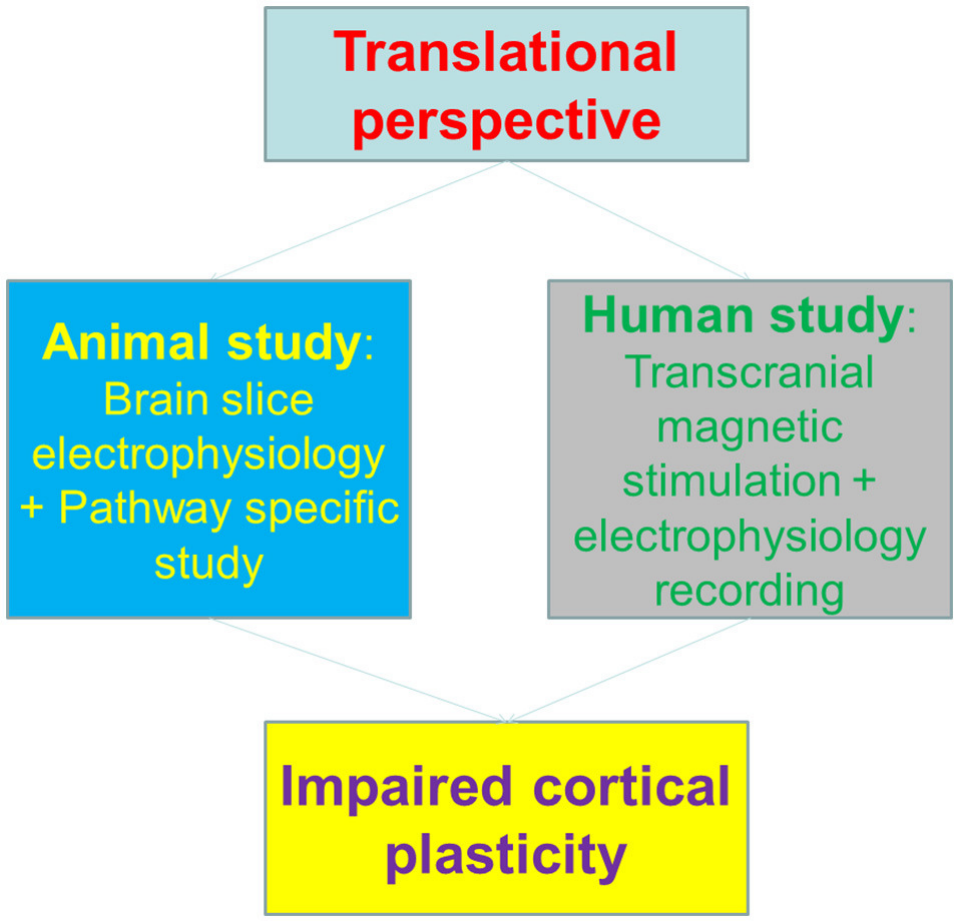
The translational perspective and working scheme of Huang et al. paper.

Motor cortex is commonly a neglected region in addiction field. However, neuroimaging findings demonstrated that craving evoked by drug-associated cues involved motor and sensory regions (Yalachkov et al., 2010). In addition, animal studies detected drug cue-associated c-Fos expression in dorsal striatum (Willuhn and Steiner, 2006). Most importantly, the compulsive drug taking behavior could share certain neural pathways as obsessive compulsive disorder (OCD), therefore motor-striatal pathway might represent a new target in drug addiction (Everitt and Robbins, 2005). Indeed, exercise therapy is proved with efficacy in addiction rehabilitation, both in animal studies and human patients (Sanchez et al., 2015). Future studies are required to further elucidate if targeting motor cortex could bring benefits in addiction rehabilitation. Interesting, in addition to methamphetamine addiction, heroin addicts also exhibited cortical plasticity deficits (Shen et al., 2017).

Cortical plasticity is affected by a number of factors, such as genetic susceptibility to activity-dependent plasticity, trophic factor expression, neurotransmitters (Li Voti et al., 2011). Besides its applications on treatment of addiction or psychiatric diseases (Shen et al., 2016; Diana et al., 2017), TMS provides the unique chance to translate previous animal findings onto human subjects, the results of which could be taken for disease state diagnosis or prognosis for therapeutic treatments. In the future, TMS dependent measurements of EEG signals could provide functional cortex mapping non-invasively, but with much higher temporal resolution than brain imaging (e.g., fMRI; Miniussi and Thut, 2010). This will largely expand our understanding in addiction related brain functional changes, and to develop potential treatment against substance abuse.

Cortical plasticity impairment, however, is not limited to addiction. Previous studies reported that schizophrenia (Fitzgerald et al., 2004; Zhou et al., 2017), depression (Duman et al., 2016), and Alzheimer's disease (Di Lorenzo et al., 2016) patients also exhibited cortical function changes and plasticity deficits. This suggested that cortical functioning or ability of cortical modulation were blunted in these diseases. It is highly plausible that certain type of molecules (e.g., GluN3A) are involved in development and progression of these diseases (Pérez-Otaño et al., 2016); it is also possible that there are different factors altered in these diseases, though converged into the commonality of plasticity deficits. In addition, the circulating BDNF or neurotransmitter levels could be similar across different cortical areas, due to the diffusion with cerebrospinal fluid, resulting in changes of both motor cortex and other cortical areas simultaneously. These possibilities are worth of future investigation.

## Author contributions

All authors listed have made a substantial, direct and intellectual contribution to the work, and approved it for publication.

### Conflict of interest statement

The authors declare that the research was conducted in the absence of any commercial or financial relationships that could be construed as a potential conflict of interest.

## References

[B1] Diana M. Raij T. Melis M. Nummenmaa A. Leggio L. Bonci A. (2017). Rehabilitating the addicted brain with transcranial magnetic stimulation. Nat. Rev. Neurosci. 18, 685–693. 10.1038/nrn.2017.113 28951609

[B2] Di Lorenzo F. Ponzo V. Bonnì S. Motta C. Negrao Serra P. C. Bozzali M.. (2016). Long-term potentiation-like cortical plasticity is disrupted in Alzheimer's disease patients independently from age of onset. Ann. Neurol. 80, 202–210. 10.1002/ana.24695 27255833

[B3] Duman R. S. Aghajanian G. K. Sanacora G. Krystal J. H. (2016). Synaptic plasticity and depression: new insights from stress and rapid-acting antidepressants. Nat. Med. 22, 238–249. 10.1038/nm.4050 26937618 PMC5405628

[B4] Etkin A. (2016). Impaired cortical plasticity in drug abuse. Sci. Transl. Med. 8:ec113 10.1126/scitranslmed.aah3544

[B5] Everitt B. J. Robbins T. W. (2005). Neural systems of reinforcement for drug addiction: from actions to habits to compulsion. Nat. Neurosci. 8, 1481–1489. 10.1038/nn1579 16251991

[B6] Fitzgerald P. B. Brown T. L. Marston N. A. Oxley T. De Castella A. Daskalakis Z. J.. (2004). Reduced plastic brain responses in schizophrenia: a transcranial magnetic stimulation study. Schizophr. Res. 71, 17–26. 10.1016/j.schres.2004.01.018 15374568

[B7] Huang X. Chen Y. Y. Shen Y. Cao X. Li A. Liu Q.. (2017). Methamphetamine abuse impairs motor cortical plasticity and function. Mol. Psychiatry 22, 1274–1281. 10.1038/mp.2017.143 28831198 PMC5582165

[B8] Huang Y. Z. Edwards M. J. Rounis E. Bhatia K. P. Rothwell J. C. (2005). Theta burst stimulation of the human motor cortex. Neuron 45, 201–206. 10.1016/j.neuron.2004.12.033 15664172

[B9] Li Voti P. Conte A. Suppa A. Iezzi E. Bologna M. Aniello M. S.. (2011). Correlation between cortical plasticity, motor learning and BDNF genotype in healthy subjects. Exp. Brain Res. 212, 91–99. 10.1007/s00221-011-2700-5 21537966

[B10] Lüscher C. Malenka R. C. (2011). Drug-evoked synaptic plasticity in addiction: from molecular changes to circuit remodeling. Neuron 69, 650–663. 10.1016/j.neuron.2011.01.017 21338877 PMC4046255

[B11] Mameli M. Bellone C. Brown M. T. Lüscher C. (2011). Cocaine inverts rules for synaptic plasticity of glutamate transmission in the ventral tegmental area. Nat. Neurosci. 14, 414–416. 10.1038/nn.2763 21336270

[B12] Miniussi C. Thut G. (2010). Combining TMS and EEG offers new prospects in cognitive neuroscience. Brain Topogr. 22, 249–256. 10.1007/s10548-009-0083-8 19241152

[B13] Pérez-Otaño I. Larsen R. S. Wesseling J. F. (2016). Emerging roles of GluN3-containing NMDA receptors in the CNS. Nat. Rev. Neurosci. 17, 623–635. 10.1038/nrn.2016.92 27558536

[B14] Sanchez V. Lycas M. D. Lynch W. J. Brunzell D. H. (2015). Wheel running exercise attenuates vulnerability to self-administer nicotine in rats. Drug Alcohol Depend. 156, 193–198. 10.1016/j.drugalcdep.2015.09.022 26433561 PMC4633318

[B15] Shen Y. Cao X. Shan C. Dai W. Yuan T. F. (2017). Heroin Addiction impairs human cortical plasticity. Biol. Psychiatry 81, e49–e50. 10.1016/j.biopsych.2016.06.013 27567311

[B16] Shen Y. Cao X. Tan T. Shan C. Wang Y. Pan J.. (2016). 10-Hz repetitive transcranial magnetic stimulation of the left dorsolateral prefrontal cortex reduces heroin cue craving in long-term addicts. Biol. Psychiatry 80, e13–e14. 10.1016/j.biopsych.2016.02.006 26995024

[B17] Willuhn I. Steiner H. (2006). Motor-skill learning-associated gene regulation in the striatum: effects of cocaine. Neuropsychopharmacology 31, 2669–2682. 10.1038/sj.npp.1300995 16395306

[B18] Yalachkov Y. Kaiser J. Naumer M. J. (2010). Sensory and motor aspects of addiction. Behav. Brain Res. 207, 215–222. 10.1016/j.bbr.2009.09.015 19751771

[B19] Zhou D. Pang F. Liu S. Shen Y. Liu L. Fang Z.. (2017). Altered motor-striatal plasticity and cortical functioning in patients with schizophrenia. Neurosci. Bull. 33, 307–311. 10.1007/s12264-016-0079-9 27838828 PMC5567505

